# Impact of exercise rehabilitation and behavioral interaction nursing on postoperative quality of life and psychological outcomes in lung cancer patients

**DOI:** 10.3389/fonc.2026.1742210

**Published:** 2026-06-17

**Authors:** Wenhui Zheng, Jinghua Ji

**Affiliations:** The First Affiliated Hospital of China Medical University, Shenyang, Liaoning, China

**Keywords:** behavioral interaction nursing, exercise rehabilitation, lung cancer, postoperative care, psychological intervention, quality of life, retrospective cohort, STROBE

## Abstract

**Background:**

Patients with lung cancer face significant physical and psychological challenges after surgery. Exercise rehabilitation and behavioral interventions show promise, but their combined association with postoperative lung cancer outcomes remains underexplored.

**Objective:**

To evaluate the association between combined exercise rehabilitation, behavioral interaction nursing, and postoperative outcomes in patients with lung cancer.

**Methods:**

This retrospective cohort study, reported according to the STROBE guidelines, included 205 patients with lung cancer (January 2023-December 2024) categorized into intervention (n=103) and control (n=102) groups based on the documented postoperative care pathway each patient received. Propensity score matching and multivariate analyses were performed to address potential confounding factors. Primary and secondary outcomes were assessed at baseline, 2, 4, and 8 weeks, and at the 5-month follow-up.

**Results:**

The intervention group demonstrated improvements in all domains. Quality of life scores (QLICP-LU) increased substantially from 62.5 ± 8.9 to 79.6 ± 6.3, representing a 27.3% improvement, versus minimal change in controls from 61.2 ± 9.1 to 60.5 ± 9.2 (between-group difference 19.1 points, P<0.001). Arterial oxygenation improved significantly (PaO_2_: 89.3 ± 5.7 vs 78.2 ± 6.1 mmHg; SaO_2_: 98.0 ± 1.3% vs. 94.5 ± 2.0%, both P<0.001). Muscle tone recovery showed substantial enhancement, with 48.2% and 55.5% improvements in the upper and lower extremities, respectively, compared to the controls (P<0.001). Systemic inflammation decreased markedly (hs-CRP level: 20.9% reduction vs. stable controls, P<0.001). Psychological outcomes revealed notable improvements: depression prevalence decreased by 62.0% (17.5% vs. 46.1%) and anxiety declined by 53.3% (24.3% vs. 52.0%, both P<0.001). Hope scores increased by 34.5%, and self-efficacy improved by 28.5% (both P<0.001). These associations persisted through the 5-month follow-up; however, residual confounding cannot be excluded from the study.

**Conclusion:**

Combined exercise rehabilitation and behavioral interaction nursing were associated with enhanced multidimensional postoperative recovery, suggesting that this approach may represent a promising comprehensive rehabilitation strategy for patients with lung cancer. Given the observational design and potential for unmeasured confounding, prospective randomized controlled trials are essential to establish causality and confirm these findings.

## Introduction

Lung cancer is the most prevalent malignant neoplasm globally, maintaining its position as the leading cause of cancer-related mortality, with approximately 1.8 million deaths annually (1, 2). Lung cancer is classified into two primary histological categories: non-small cell lung carcinoma (NSCLC) and small cell lung carcinoma (SCLC), which collectively account for over 95% of documented cases (3). Recent epidemiological surveillance has demonstrated that lung cancer continues to dominate both incidence and mortality statistics, with 2.5 million new cases and 1.8 million fatalities reported in 2022 alone (4). This persistent epidemiological burden underscores the critical importance of comprehensive therapeutic approaches that extend beyond primary tumor management to encompass holistic patient care and long-term survival outcomes.

Contemporary lung cancer treatment paradigms have evolved substantially, incorporating multimodal therapeutic strategies, including surgical resection, stereotactic radiation therapy, systemic chemotherapy, immunotherapy, and precision molecular interventions (5–7). Despite these remarkable advances in therapeutic options, surgical resection remains the cornerstone of curative treatment for early stage disease and selected locally advanced cases, offering the most favorable long-term survival outcomes (7). However, the postoperative recovery period presents substantial multidimensional challenges that significantly affect patient recovery trajectories, functional outcomes and overall quality of life. The physiological stress imposed by thoracic surgery induces profound alterations in respiratory mechanics, cardiovascular function, and neuromuscular coordination, while simultaneously triggering complex psychological responses that frequently manifest as anxiety, depression, and fear of disease recurrence (8, 9).

The intricate interplay between physical and psychological factors during postoperative recovery has been increasingly recognized as a critical determinant of both short-term recovery success and long-term survival outcomes (10, 11). Patients commonly experience diminished functional capacity, compromised respiratory efficiency, progressive muscle deconditioning, and reduced exercise tolerance, which collectively contribute to a decreased quality of life and prolonged rehabilitation timelines. Simultaneously, the psychological burden associated with cancer diagnosis, surgical intervention, and uncertain prognosis often precipitates significant emotional distress, which is characterized by heightened anxiety levels, depressive symptoms, and compromised adaptive coping mechanisms (12, 13). These psychological manifestations are further exacerbated by multifaceted socioeconomic factors, including financial strain from treatment costs, altered family dynamics, work-related concerns, and educational background disparities, creating a complex biopsychosocial challenge that demands comprehensive, evidence-based intervention strategies.

Traditional postoperative care protocols, while effectively addressing immediate surgical complications and basic physiological recovery needs, often fail to adequately address the complex, interconnected biopsychosocial requirements of lung cancer patients during their recovery (14). The fundamental limitation of conventional approaches lies in their fragmented nature, typically focusing primarily on physiological recovery parameters while neglecting the psychological, social, and spiritual dimensions of patient well-being. This significant gap in comprehensive care has prompted the development and investigation of integrated intervention models that simultaneously target physical rehabilitation, psychological support, and social reintegration to optimize overall recovery outcomes and enhance the quality of long-term survival.

Exercise rehabilitation has emerged as a fundamental, evidence-based component of contemporary cancer survivorship care, with studies demonstrating its association with improved cardiopulmonary function, enhanced muscle strength and endurance, optimized immune system, and supported overall physical well-being (15, 16). Structured progressive exercise programs facilitate improved circulation patterns, reduce venous thromboembolism risks, enhance pulmonary function and gas exchange efficiency, and prevent common postoperative complications, such as atelectasis, pneumonia, and cardiovascular deconditioning (17). Furthermore, regular physical activity has been consistently associated with reduced systemic inflammation, improved immune function markers, enhanced mood regulation, and improved quality of life metrics across diverse cancer populations (18).

Behavioral interaction nursing represents an innovative and comprehensive approach to psychosocial support that integrates evidence-based psychological interventions with structured, patient-centered nursing care protocols (19). This methodology encompasses individualized psychological counseling utilizing cognitive-behavioral therapy principles, peer support facilitation through structured group interactions, and comprehensive patient and family education programs designed to address the complex emotional, informational, and social needs of cancer patients throughout their recovery journey (20). This approach emphasizes patient empowerment through systematic knowledge acquisition, adaptive coping skill development, and social support network strengthening, ultimately fostering psychological resilience and promoting adaptive recovery patterns that extend well beyond the acute treatment phase.

The present study aimed to examine the association between participation in a combined exercise rehabilitation and behavioral interaction nursing program and postoperative outcomes among patients with lung cancer using a retrospective cohort methodology. We hypothesized that patients who received comprehensive intervention would demonstrate superior quality of life, psychological well-being, and physiological recovery compared with those who received standard postoperative care alone.

## Methods

### Study design and ethical considerations

This retrospective cohort study was conducted according to the Strengthening the Reporting of Observational Studies in Epidemiology (STROBE) guidelines for observational research (21). This study was conducted at The First Affiliated Hospital of China Medical University, reviewing the medical records of patients treated between January 2023 and December 2024. This comprehensive rehabilitation program was prospectively implemented as a clinical service enhancement program in January 2023. The present study outcomes constitute a retrospective analysis of clinical outcomes derived from medical records, comparing patients who participated in the program with those who received standard care during the same period. This retrospective analysis was not prospectively powered; the sample size was determined based on the number of patients who met the eligibility criteria during the 24-month study period.

The study protocol was approved by the Institutional Review Board (IRB Protocol No. 2023-IRB-028), with a waiver of informed consent for the retrospective medical record review. This study adhered to the ethical principles outlined in the Declaration of Helsinki and the institutional research ethics standards for retrospective research. All data were de-identified and maintained confidentially in accordance with HIPAA regulations.

### Intervention setting and context

The comprehensive rehabilitation program was delivered within the inpatient rehabilitation unit (20 beds) and outpatient thoracic oncology clinic at our institution’s cancer center. The program was developed by a multidisciplinary team that included thoracic surgeons, pulmonologists, rehabilitation medicine specialists, clinical psychologists, and oncology nursing leadership. According to institutional records, intervention delivery was carried out by a dedicated team comprising four certified rehabilitation therapists (each with >5 years of oncology rehabilitation experience), two licensed clinical psychologists with specialized oncology training, and six oncology nurses who had completed a 40-hour standardized training program before program implementation.

The decision to enroll patients in the comprehensive rehabilitation program (versus standard care) was made collaboratively by the surgical team, rehabilitation consultants, and patients/families based on clinical factors, including postoperative recovery trajectory, perceived rehabilitation potential, patient interest and availability, bed availability in the rehabilitation unit, and timing of discharge planning. This non-random allocation process may have introduced selection bias, as clinicians may have preferentially recommended the program to patients perceived as more motivated or with better prognoses than others.

### Participant selection and characteristics

#### Participant identification

A total of 267 consecutive patients who underwent surgical resection for lung cancer were identified through a retrospective medical record review during the 24-month study period. Electronic health records were systematically searched using ICD-10 diagnosis codes and surgical procedure codes to identify all potentially eligible patients whose records documented lung cancer surgery during the specified time frame.

Inclusion criteria comprised: histologically confirmed primary bronchogenic carcinoma of any cell type verified through tissue biopsy or cytological examination; World Health Organization (WHO) performance status ≤2 indicating adequate functional capacity; successful documented completion of radical lung cancer surgery with curative intent as determined by the surgical team; hemodynamically stable postoperative condition permitting safe participation in structured physical and behavioral interventions; preoperative and postoperative cardiac and pulmonary function parameters within acceptable ranges for exercise participation as documented in medical records; cognitive capacity to understand program requirements; documented participation in either the comprehensive rehabilitation program or standard postoperative care pathway.

Exclusion criteria included severe cardiopulmonary dysfunction precluding safe exercise participation as determined by cardiologist or pulmonologist evaluation; hepatic or renal dysfunction with laboratory values exceeding twice the upper limit of normal ranges; significant motor dysfunction, neurological disorders, or psychiatric illness that would substantially interfere with intervention compliance or safety; concurrent participation in other structured psychological, behavioral, or exercise intervention programs that could confound study results; cognitive impairment or language barriers limiting comprehension of program components; documented patient or designated decision-maker refusal to participate in any aspect of the research protocol; anticipated survival of less than 6 months based on oncological assessment; and planned adjuvant therapy initiation within the 8-week intervention period.

#### Group assignment

Patients were categorized into intervention or control groups based on a retrospective review of their documented postoperative care pathways. The intervention group consisted of patients whose medical records documented participation in the structured 8-week exercise rehabilitation and behavioral interaction nursing program. The control group comprised patients who received standard postoperative care without documented participation in the comprehensive rehabilitation program during the same period. This group assignment was determined by clinical practice patterns rather than random allocation; therefore, baseline differences between the groups may reflect selection factors rather than chance.

#### Propensity score analysis and confounding control

To address potential confounding factors from non-random group assignment, we conducted a propensity score analysis. Propensity scores representing the probability of receiving the intervention were estimated using multivariable logistic regression with the following covariates: age, sex, body mass index, tumor stage (I-II vs. III), histological type (NSCLC vs. SCLC), baseline QLICP-LU score, baseline SAS score, baseline SDS score, baseline PaO_2_, and baseline mMRC dyspnea score.

We employed two complementary approaches (1): propensity score matching using 1:1 nearest-neighbor matching without replacement with a caliper width of 0.2 standard deviations of the logit of the propensity score, and (2) inverse probability of treatment weighting (IPTW) for sensitivity analysis. It should be noted that propensity score methods can only adjust for measured confounders and cannot account for unmeasured or residual confounding factors. Covariate balance after matching was assessed using standardized mean differences, with values <0.1 indicating adequate balance. Full propensity score diagnostics, model specifications, E-value calculations, and IPTW weight truncation procedures are reported in **Supplementary Appendix S2**.

#### Data abstraction and quality assurance

Trained research assistants extracted clinical data from electronic medical records using standardized data-collection forms. The abstractors were not involved in clinical care delivery. A senior investigator reviewed 15% of the abstracted records for accuracy, and discrepancies were resolved through a consensus review. The inter-rater reliability for the key variables exceeded 95%.

### Intervention protocols

#### Control group management

The control group (n=102) received standardized postoperative care according to institutional evidence-based protocols, which included systematic vital sign monitoring every 4–6 hours during hospitalization, comprehensive surgical wound care with daily assessment and appropriate dressing changes, multimodal pain management utilizing validated protocols combining pharmacological and non-pharmacological approaches, structured nutritional counseling by registered dietitians focusing on protein optimization and caloric adequacy, and conventional rehabilitation guidance emphasizing gradual mobilization, basic breathing exercises, and activities of daily living progression. Standardized discharge planning included detailed written instructions, scheduled follow-up appointments with the surgical team, and 24-hour contact information for urgent concerns.

#### Intervention group protocol

The intervention group (n=103) received all standard care components plus a comprehensive, individually tailored, 8-week structured intervention program combining progressive exercise rehabilitation with behavioral interaction nursing, initiated according to clinical records on the third postoperative day following surgical team clearance and confirmation of hemodynamic stability. The program comprised three principal components, summarized below: comprehensive exercise prescription details, session-by-session protocols, and progression criteria are provided in **Supplementary Appendix S1**.

#### Exercise rehabilitation component

The exercise rehabilitation program encompassed three sequential components. Respiratory function training included diaphragmatic breathing exercises with visual biofeedback, pursed-lip breathing techniques, and incentive spirometry, delivered in 15-minute sessions twice daily, with initial supervision by respiratory therapists and progression to independent practice based on competency assessments. Upper extremity rehabilitation consisted of progressive resistance training targeting shoulder girdle musculature following the American College of Sports Medicine guidelines for cancer survivors, beginning at 40–50% of the estimated one-repetition maximum with weekly progression as tolerated. Lower extremity and functional mobility training commenced with supervised bedside standing and progressed through assisted ambulation to independent walking, with exercise intensity guided by heart rate monitoring, the modified Borg scale, and continuous assessment of oxygen saturation. Sessions were conducted twice daily for 15–30 min. Full exercise prescription details, including specific sets, repetitions, resistance levels, and individualized progression criteria, are provided in the **Supplementary Appendix S1**.

#### Behavioral interaction nursing component

The behavioral intervention comprised three integrated elements: individual psychological counseling delivered by licensed clinical psychologists in weekly 30–45-minute sessions utilizing a structured 8-session cognitive-behavioral therapy protocol adapted for cancer patients. Peer support group activities consisted of twice-weekly structured group sessions of 6–8 participants, facilitated by trained mental health professionals, incorporating discussions of shared experiences, collaborative problem-solving, and peer mentorship. Comprehensive patient and family education was delivered through weekly 45–60-minute sessions addressing postoperative self-care, pain management, nutritional optimization, symptom recognition, and recovery expectations. Detailed session structures and weekly curricula are provided in the **Supplementary Appendix S1**.

### Intervention fidelity monitoring

As part of standard clinical quality improvement during prospective program delivery, fidelity was monitored through therapist completion of standardized session checklists, monthly supervision meetings with program leadership, and quarterly chart audits reviewing 10% of participant records. These clinical quality data were retrospectively abstracted for research purposes. Overall protocol adherence, defined as completion of ≥80% of scheduled sessions, was 89.3% in the intervention group.

### Outcome measures and assessment protocols

#### Primary outcomes

Quality of life assessment: The Quality-of-Life Instrument for Chronic Diseases-Lung Cancer (QLICP-LU) served as the primary QoL measure. This validated 29-item instrument, specifically designed for lung cancer populations, encompasses four domains: physical function (8 items), psychological function (7 items), social function (7 items), and common symptoms (7 items). Scoring was performed using a 5-point Likert scale, with higher scores indicating a better quality of life. The instrument demonstrates excellent psychometric properties, with a Cronbach’s alpha >0.90, and a minimal clinically important difference of 8 points established in validation studies.

Psychological Well-being Evaluation: Psychological status was comprehensively evaluated using validated instruments, including the Self-Rating Anxiety Scale (SAS) and Self-Rating Depression Scale (SDS). Both instruments contain 20 items scored on a 4-point Likert scale with established cutoff values for clinical significance (SAS≥50 indicating clinically significant anxiety; SDS≥53 indicating clinically significant depression). These scales demonstrate strong psychometric properties in cancer populations, with test-retest reliability coefficients >0.85, and have been validated for use in Chinese cancer populations.

#### Secondary outcomes

Physiological parameters: Detailed Laboratory Protocols Arterial blood gas analysis was used to measure the partial pressure of oxygen (PaO_2_) and oxygen saturation (SaO_2_) using standardized arterial puncture techniques. Samples were analyzed using calibrated blood gas analyzers (ABL90 FLEX, Radiometer) with daily calibration and verification. High-sensitivity C-reactive protein (hs-CRP) levels were quantified using an automated immunoassay (ADVIA Centaur XP, Siemens) with a coefficient of variation <3%.

Anthropometric and functional assessments: Body mass index (BMI) was calculated using standardized anthropometric measurements obtained using calibrated scales and stadiometers according to the WHO Health Organization protocols (22). Muscle tone was assessed using the Medical Research Council (MRC) manual muscle testing scale, which is a validated 0–5-point ordinal scale. In this study, scores were reported as deviations from normal (5 minus the observed score), such that lower values indicated better function. Assessments were performed by trained physical therapists (n=4), who demonstrated inter-rater reliability of ICC = 0.91. The modified Medical Research Council (mMRC) dyspnea scale was used to assess breathlessness severity during activities of daily living. Exercise self-efficacy was measured using the Self-Efficacy for Exercise Scale (SEE-C), and hope levels were quantified using the Herth Hope Index (HHI), both validated instruments with established psychometric properties for cancer population.

### Data collection procedures and timeline

All assessment data were extracted from the standardized clinical documentation. Baseline assessments were completed within 24 h before the intervention initiation. Subsequent evaluations occurred at approximately 2, 4, and 8-weeks post-intervention initiation (± 3 days), with extended follow-up assessments at 1, 2, 3, 4, and 5-months post-intervention completion (± 7 days). Each comprehensive assessment session lasted approximately 90–120 minutes and included all primary and secondary outcome measures with standardized rest periods to minimize fatigue effects. All physiological measurements were obtained during standardized morning hours (8:00-10:00 AM) following overnight fasting, when applicable, to minimize circadian variation effects. Data were entered into a secure web-based electronic data capture system (REDCap) with real-time data validation and audit trails. Double data entry was performed for 20% of records to ensure accuracy.

### Statistical analysis plan

#### Comprehensive statistical methodology

Statistical analyses were performed using SPSS version 29.0 and R software version 4.3.0, with significance levels set at α<0.05 for primary outcomes and α<0.01 for secondary outcomes to partially address multiple secondary comparisons.

Missing Data: Missing data patterns were examined and found to be <5% for the primary outcomes at all time points. Primary analyses were conducted using the available data, and sensitivity analyses using multiple imputations (m=20 imputations) under missing-at-random assumptions were conducted to assess robustness (details in **Supplementary Appendix S2**).

Baseline characteristics were compared using independent t-tests for normally distributed continuous variables, Mann-Whitney U tests for non-normally distributed continuous variables, and chi-square or Fisher’s exact tests for categorical variables. Distribution normality was assessed using the Shapiro-Wilk test, Q-Q plots, and histogram inspection.

Primary analyses utilized linear mixed-effects models to examine group differences over time, accounting for within-subject correlations using random intercepts. The models included fixed effects for the treatment group, time, and group × time interactions. The covariates included in the adjusted models were age, sex, tumor stage, and baseline values of the outcome variables.

Effect sizes were calculated using Cohen’s d with 95% confidence intervals and were interpreted as small (0.2), medium (0.5), and large (0.8).

Propensity score–matched analyses were used to assess between-group differences using paired t-tests for continuous outcomes and McNemar’s test for categorical outcomes. IPTW-adjusted analyses used weighted regression models. The full model specifications and sensitivity analysis procedures are reported in **Supplementary Appendix S2**.

## Results

### Participant flow and baseline characteristics

**Figure 1:**
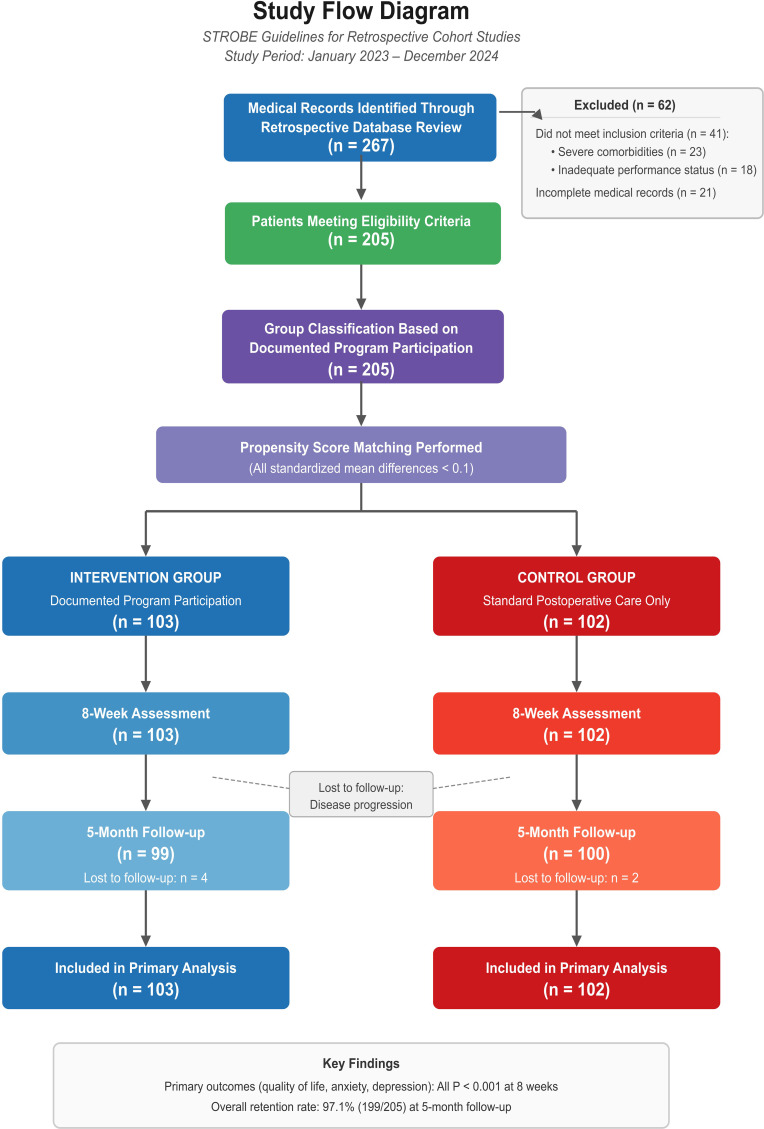
Study flow diagram according to STROBE guidelines for retrospective cohort studies. Patients classified based on documented participation in structured intervention program. Propensity score matching performed to address potential selection bias.

Between January 2023 and December 2024, 267 patients were systematically assessed for study eligibility through a retrospective review of medical records. Of these, 62 patients were excluded due to failure to meet the inclusion criteria (n=41), primarily related to severe comorbidities or inadequate performance status, and incomplete medical records (n=21). The final study cohort comprised 205 participants who were categorized into intervention (n=103) and control (n=102) groups based on the documented care received (**Figure 1**).

All 205 identified participants had complete 8-week data for the primary outcomes, and 199 participants (97.1%) had complete data through the 5-month extended follow-up assessment, with six participants lost to follow-up due to disease progression requiring intensive adjuvant therapy that precluded continued outcome assessment.

Comprehensive baseline demographic and clinical characteristics demonstrated reasonable between-group similarities across most measured parameters (**Table 1**). The study population consisted predominantly of older adults with a mean age of 62.4 ± 4.3 years, representing the typical lung cancer demographics. Sex distribution was balanced (54.1% male), and tumor staging profiles showed appropriate representation of early and locally advanced disease stage. Importantly, most physiological, functional, and psychological parameters showed no statistically significant baseline differences (all P>0.05); however, the intervention group showed a trend toward better baseline upper limb muscle tone scores (P = 0.106), suggesting a possible selection bias in patients with better functional status in the intervention group. This potential selection bias was addressed using propensity score matching analysis.

**Table 1:** Baseline demographic and clinical characteristics.

Characteristic	Intervention group (n=103)	Control group (n=102)	Test statistic	P-value
Demographics				
Age (years), mean ± SD	62.1 ± 4.4	62.7 ± 4.2	t = 1.02	0.312
Male gender, n (%)	56 (54.4)	55 (53.9)	χ² = 0.005	0.942
Anthropometric measures				
Height (m), mean ± SD	1.65 ± 0.08	1.64 ± 0.08	t = 0.89	0.376
Weight (kg), mean ± SD	68.7 ± 9.2	67.8 ± 8.8	t = 0.72	0.473
BMI (kg/m²), mean ± SD	25.1 ± 3.1	25.2 ± 2.9	t = 0.24	0.808
Tumor characteristics				
Stage I-II, n (%)	62 (60.2)	61 (59.8)	χ² = 0.003	0.958
Stage III, n (%)	41 (39.8)	41 (40.2)	–	–
NSCLC histology, n (%)	89 (86.4)	87 (85.3)	χ² = 0.054	0.816
Physiological parameters				
SaO_2_ (%), mean ± SD	96.2 ± 1.6	95.8 ± 1.7	t = 1.75	0.082
PaO_2_ (mmHg), mean ± SD	82.3 ± 6.2	80.9 ± 6.1	t = 1.64	0.103
hs-CRP (mg/L), mean ± SD	5.31 ± 2.18	5.59 ± 2.41	t = 0.89	0.376
Functional assessments				
Upper limb muscle tone score	1.14 ± 0.43	1.29 ± 0.39	t = 2.67	0.106
Lower limb muscle tone score	1.19 ± 0.36	1.24 ± 0.42	t = 0.91	0.365
mMRC dyspnea score	1.43 ± 0.61	1.49 ± 0.59	t = 0.71	0.477
Psychological measures				
SAS score, mean ± SD	48.4 ± 6.9	49.1 ± 7.3	t = 0.72	0.474
SDS score, mean ± SD	50.5 ± 7.6	51.2 ± 8.1	t = 0.64	0.521
HHI score, mean ± SD	28.7 ± 5.8	28.1 ± 6.2	t = 0.73	0.468
Quality of life				
QLICP-LU score, mean ± SD	62.5 ± 8.9	61.2 ± 9.1	t = 1.03	0.304
SEE-C score, mean ± SD	35.8 ± 4.9	35.1 ± 5.2	t = 0.98	0.329

BMI, body mass index; SaO_2_, arterial oxygen saturation; PaO_2_, partial pressure of oxygen; hs-CRP, high-sensitivity C-reactive protein; mMRC, modified Medical Research Council; SAS, Self-Rating Anxiety Scale; SDS, Self-Rating Depression Scale; HHI, Herth Hope Index; QLICP-LU, Quality of Life Instrument for Chronic Diseases-Lung Cancer; SEE-C, Self-Efficacy for Exercise Scale; NSCLC, non-small cell lung cancer.

### Propensity score analysis results

The propensity score model demonstrated adequate discrimination (C-statistic = 0.68). Following 1:1 nearest-neighbor matching, 89 matched pairs were identified in each group. Covariate balance improved substantially after matching, with all standardized mean differences <0.1, indicating an adequate balance of the measured confounders (**Supplementary Table 1**). IPTW analysis included all 205 participants with appropriate weight truncation at the 1st and 99th percentiles of the weight distribution.

### Primary outcome: quality of life and psychological well-being

#### Unadjusted analyses

**Figure 2:**
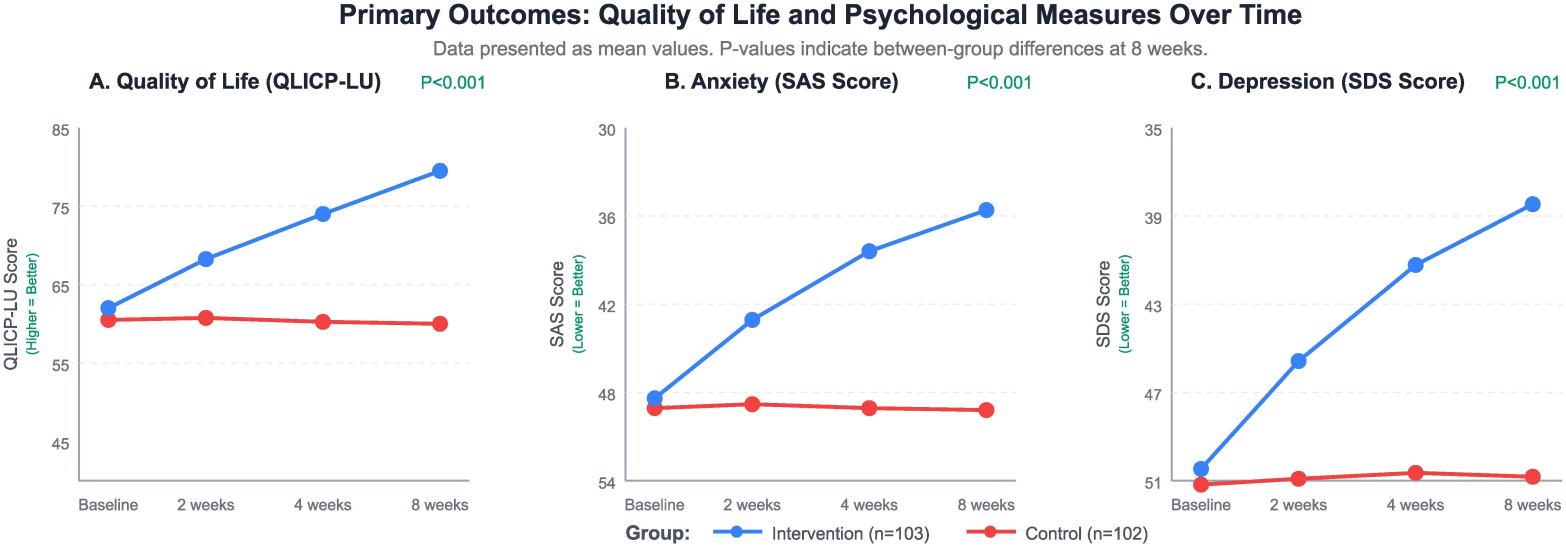
Primary outcomes over time in lung cancer patients receiving combined exercise rehabilitation and behavioral interaction nursing. Quality of life (QLICP-LU), anxiety (SAS), and depression (SDS) scores were measured at baseline, 2-, 4-, and 8-weeks post-intervention initiation. Data are presented as mean values. **(A)** shows the QLICP-LU scores, where higher values indicate a better quality of life. **(B, C)** show the SAS and SDS scores, respectively, with the Y-axis inverted so that improvement (lower scores) appears as an upward movement on the graph. The intervention group (blue, n=103) received exercise rehabilitation plus behavioral interaction nursing, the and control group (red, n=102) received standard postoperative care only. Between-group differences were analyzed using linear mixed-effects models with adjustments for baseline values. All P<0.001 at eight weeks. QLICP-LU, Quality of Life Instrument for Chronic Diseases-Lung Cancer; SAS, Self-Rating Anxiety Scale; SDS, Self-Rating Depression Scale.

Participation in the intervention was associated with substantial differences in quality-of-life measures, which represented the primary endpoint of the study. Quality of life assessment using the QLICP-LU instrument revealed progressive and clinically meaningful improvements in the intervention group throughout the 8-week observation period, while the control group scores remained stable (**Figure 2A**). At the primary endpoint of 8 weeks, intervention group QLICP-LU scores increased from baseline values of 62.5 ± 8.9 to 79.6 ± 6.3, representing a substantial 27.3% enhancement and a large effect size (Cohen’s d = 1.87,95% CI: 1.54-2.20). In contrast, the control group scores showed minimal variation (61.2 ± 9.1 to 60.5 ± 9.2), resulting in a clinically meaningful between-group difference of 19.1 points (95% CI: 14.8-23.4, P<0.001), which substantially exceeded the established minimal clinically important difference of 8 points for this instrument.

Psychological well-being assessments revealed substantial between-group differences across all measured domains in the intervention group, with particularly pronounced reductions in anxiety and depressive symptoms among the intervention participants (**Figures 2B, C**). Self-Rating Anxiety Scale scores demonstrated progressive improvement from baseline values of 48.4 ± 6.9 to 35.6 ± 5.2 at 8 weeks in the intervention group, representing a 26.4% reduction that exceeded the minimal clinically important difference (**Table 2**). Control group SAS scores remained stable throughout the study period (49.1 ± 7.3 to 49.2 ± 7.3), resulting in a significant between-group difference of 13.6 points (95% CI: 10.2-17.0, P<0.001) with a large effect size (Cohen’s d = 2.06,95% CI: 1.72-2.40).

**Table 2:** Psychological assessment and quality of life outcomes over time.

Measure	Time point	Intervention group (n=103)	Control group (n=102)	Between-group difference (95% CI)	P-value	Effect size (Cohen’s d, 95% CI)
SAS score						
	Baseline	48.4 ± 6.9	49.1 ± 7.3	-0.7 (-2.8 to 1.4)	0.474	0.10 (-0.17 to 0.37)
	2 weeks	43.0 ± 6.3	48.8 ± 7.1	-5.8 (-7.8 to -3.8)	0.002	0.87 (0.58-1.15)
	4 weeks	38.4 ± 5.9	49.0 ± 7.2	-10.6 (-12.8 to -8.4)	0.001	1.61 (1.30-1.92)
	8 weeks	35.6 ± 5.2	49.2 ± 7.3	-13.6 (-17.0 to -10.2)	<0.001	2.06 (1.72-2.40)
SDS score						
	Baseline	50.5 ± 7.6	51.2 ± 8.1	-0.7 (-3.1 to 1.7)	0.521	0.09 (-0.18 to 0.36)
	2 weeks	45.6 ± 6.9	50.9 ± 8.0	-5.3 (-7.6 to -3.0)	0.005	0.71 (0.43-0.99)
	4 weeks	41.2 ± 6.2	50.6 ± 8.1	-9.4 (-11.8 to -7.0)	0.001	1.29(0.98-1.60)
	8 weeks	38.5 ± 5.4	50.8 ± 8.1	-12.3 (-15.9 to -8.7)	<0.001	1.93(1.60-2.26)
HHI score						
	Baseline	28.7 ± 5.8	28.1 ± 6.2	0.6 (-1.2 to 2.4)	0.468	0.10 (-0.17 to 0.37)
	2 weeks	32.2 ± 5.3	28.2 ± 6.2	4.0 (2.2 to 5.8)	0.004	0.69 (0.41-0.97)
	4 weeks	35.9 ± 5.0	28.0 ± 6.2	7.9 (6.2 to 9.6)	0.001	1.39 (1.08-1.70)
	8 weeks	38.6 ± 4.7	28.1 ± 6.3	10.5 (7.8 to 13.2)	<0.001	1.87 (1.54-2.20)
QLICP-LU score						
	Baseline	62.5 ± 8.9	61.2 ± 9.1	1.3 (-1.5 to 4.1)	0.304	0.14 (-0.13 to 0.41)
	2 weeks	68.6 ± 8.0	61.3 ± 9.1	7.3 (4.7 to 9.9)	0.001	0.84 (0.56-1.13)
	4 weeks	74.2 ± 7.2	60.9 ± 9.1	13.3 (10.8 to 15.8)	0.001	1.60 (1.29-1.91)
	8 weeks	79.6 ± 6.3	60.5 ± 9.2	19.1 (14.8 to 23.4)	<0.001	1.87(1.54-2.20)

SAS, Self-Rating Anxiety Scale; SDS, Self-Rating Depression Scale; HHI, Herth Hope Index; QLICP-LU, Quality of Life Instrument for Chronic Diseases-Lung Cancer. Data presented as mean ± SD. CI, confidence interval.

Depressive symptomatology, assessed using the Self-Rating Depression Scale, demonstrated comparable patterns, with intervention group scores declining from 50.5 ± 7.6 to 38.5 ± 5.4, in contrast to stable control group values (51.2 ± 8.1 to 50.8 ± 8.1). This 23.8% reduction in depression scores (between-group difference: 12.3 points, 95% CI: 8.7-15.9, P<0.001) corresponded to a large effect size (Cohen’s d = 1.93, 95% CI: 1.60-2.26).

**Figure 3:**
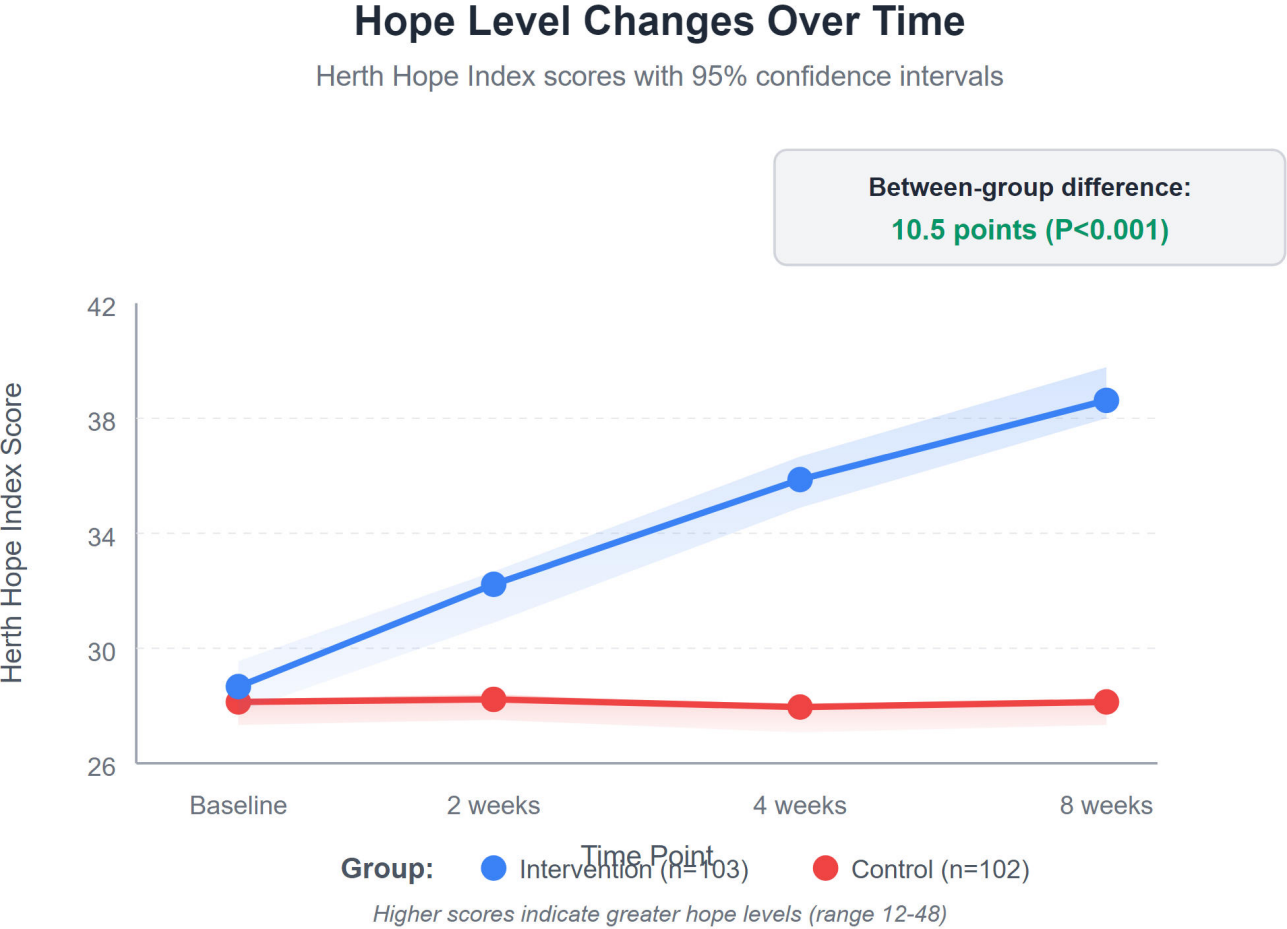
Hope level improvements measured by Herth Hope Index. Hope scores were measured at baseline, 2, 4, and 8 weeks using the validated Herth Hope Index (HHI). Data are presented as mean ± SE with 95% confidence intervals (shaded areas). Higher scores indicate greater hope levels (possible range 12-48). Between-group difference at 8 weeks: 10.5 points (95% CI: 7.8-13.2, P<0.001), representing a 34.5% improvement in the intervention group. The intervention group demonstrated progressive improvement throughout the study period, while the control group scores remained stable.

Hope assessment using the Herth Hope Index revealed substantial enhancement in the intervention participants (**Figure 3**), with progressive score increases from 28.7 ± 5.8 at baseline to 38.6 ± 4.7 at 8 weeks, while the control participants demonstrated minimal change (28.1 ± 6.2 to 28.1 ± 6.3). This 34.5% improvement in hope scores (between-group difference: 10.5 points, 95% CI: 7.8-13.2, P<0.001) exceeded the established thresholds for meaningful change and showed a strong correlation with quality-of-life improvements (Pearson r = 0.74, P<0.001).

#### Propensity score-matched analyses

In propensity score-matched analyses (n=89 pairs), the between-group differences remained statistically significant, although somewhat attenuated compared to the unadjusted analyses (**Table 3**). The matched between-group difference in QLICP-LU at 8 weeks was 17.2 points (95% CI: 12.4-22.0, P<0.001), with a matched effect size of Cohen’s d = 1.68. For SAS, the matched difference was 12.1 points (95% CI: 8.3-15.9, P<0.001); for SDS, 10.8 points (95% CI: 7.1-14.5, P<0.001). These findings suggest that the observed associations persisted after adjusting for measured confounders, although propensity score methods cannot account for unmeasured confounding, and these results should not be interpreted as establishing causality. The modest attenuation of effect sizes following matching suggests that the measured covariates partially, but not fully, accounted for the observed differences.

**Table 3:** Propensity score-matched analysis results for primary outcomes at 8 weeks.

Outcome	Matched intervention (n=89)	Matched control (n=89)	Matched difference (95% CI)	P-value	Effect size (Cohen’s d, 95% CI)
QLICP-LU	78.9 ± 6.5	61.7 ± 9.0	17.2 (12.4-22.0)	<0.001	1.68 (1.34-2.02)
SAS	36.1 ± 5.4	48.2 ± 7.1	-12.1 (-15.9 to -8.3)	<0.001	1.89 (1.54-2.24)
SDS	39.2 ± 5.6	50.0 ± 7.8	-10.8 (-14.5 to -7.1)	<0.001	1.56 (1.23-1.89)
HHI	38.1 ± 4.9	28.4 ± 6.1	9.7 (7.1-12.3)	<0.001	1.73 (1.39-2.07)

Propensity score matching performed using 1,1 nearest-neighbor matching without replacement (caliper = 0.2 SD). Data presented as mean ± SD. Effect sizes modestly attenuated compared to unadjusted analyses, suggesting partial confounding by measured covariates.

IPTW-adjusted analyses yielded similar results, with the IPTW-adjusted between-group difference in QLICP-LU of 18.3 points (95% CI: 13.9-22.7, P<0.001), supporting the robustness of the findings across analytical approaches.

### Secondary outcomes: physiological parameters and functional recovery

**Figure 4:**
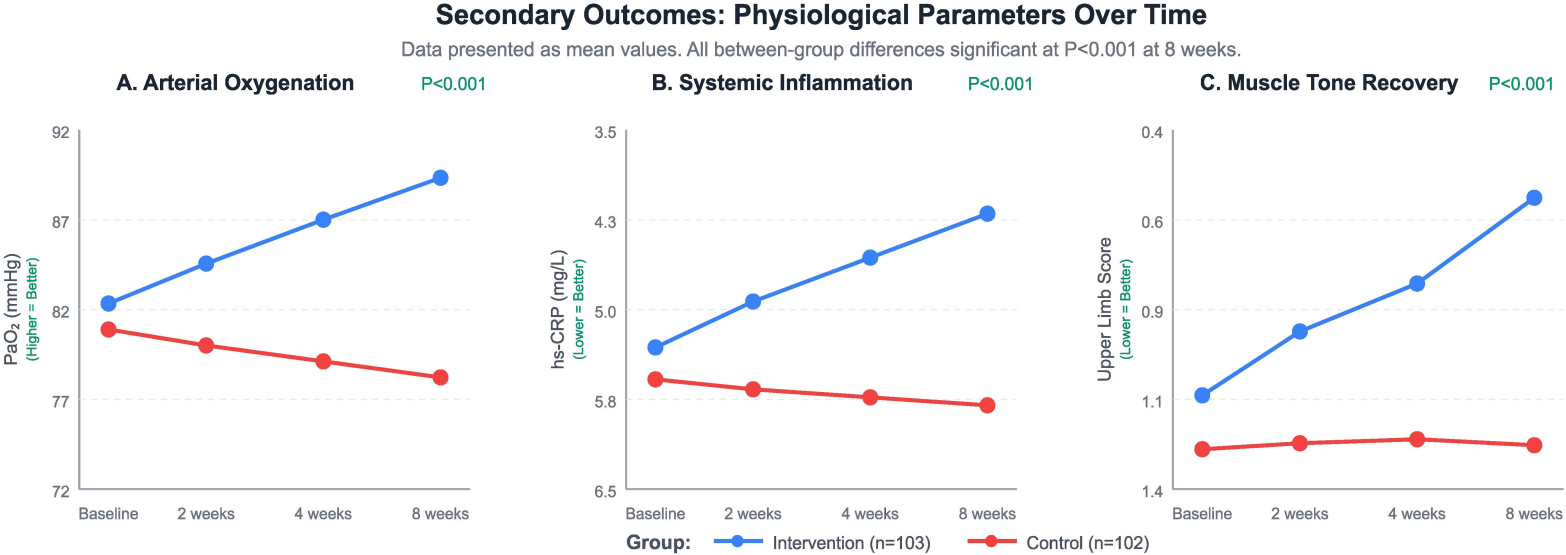
Secondary physiological outcomes demonstrating intervention efficacy. Arterial oxygenation (PaO_2_), systemic inflammation (hs-CRP), and upper limb muscle tone recovery were measured at baseline, 2, 4, and 8 weeks. Data are presented as mean values. **(A)** shows the PaO_2_, where higher values indicate better oxygenation. **(B, C)** show hs-CRP and muscle tone scores, respectively, with the Y-axis inverted so that improvement (lower values) appears as an upward movement on the graph. Lower muscle tone scores indicate improved function, and lower hs-CRP levels indicate reduced inflammation. All between-group differences were significant at P<0.001 at 8 weeks using linear mixed-effects models. PaO_2_, partial pressure of arterial oxygen; hs-CRP, high-sensitivity C-reactive protein.

Participation in the intervention was associated with notable differences in multiple physiological parameters (**Figure 4**), with progressive improvements observed throughout the 8-week observation period, reflecting the benefits of exercise training and the comprehensive care approach. Longitudinal changes in muscle tone and functional parameters are summarized in **Table 4**. Muscle tone recovery, assessed using standardized manual muscle testing protocols, represented one of the most striking secondary findings, with both upper and lower extremity assessments showing significant between-group differences emerging as early as two weeks and continuing to diverge until the end of the study (**Table 4**).

**Table 4:** Longitudinal changes in muscle tone and functional parameters.

Parameter	Time point	Intervention group (n=103)	Control group (n=102)	Between-group difference (95% CI)	P-value	Effect size (Cohen’s d, 95% CI)
Upper limb muscle tone						
	Baseline	1.14 ± 0.43	1.29 ± 0.39	-0.15 (-0.28 to -0.02)	0.106	0.37(0.09-0.64)
	2 weeks	0.96 ± 0.34	1.27 ± 0.38	-0.31 (-0.42 to -0.20)	0.028	0.87(0.58-1.15)
	4 weeks	0.83 ± 0.32	1.26 ± 0.37	-0.43 (-0.54 to -0.32)	0.003	1.24(0.94-1.54)
	8 weeks	0.59 ± 0.26	1.28 ± 0.38	-0.69 (-0.89 to -0.49)	<0.001	1.76 (1.44-2.08)
Lower limb muscle tone						
	Baseline	1.19 ± 0.36	1.24 ± 0.42	-0.05 (-0.16 to 0.06)	0.365	0.13(-0.14 to 0.40)
	2 weeks	0.89 ± 0.31	1.23 ± 0.41	-0.34 (-0.44 to -0.24)	0.014	0.93(0.64-1.22)
	4 weeks	0.76 ± 0.29	1.22 ± 0.40	-0.46 (-0.56 to -0.36)	0.002	1.32(1.01-1.63)
	8 weeks	0.53 ± 0.23	1.21 ± 0.41	-0.65 (-0.84 to -0.46)	<0.001	1.94(1.61-2.27)

Lower scores indicate an improved muscle tone. Data are presented as mean ± SD. CI, confidence interval.

Upper limb muscle tone scores in the intervention group improved from baseline values of 1.14 ± 0.43 to 0.59 ± 0.26 at 8 weeks, representing a 48.2% improvement. The control group scores remained essentially unchanged throughout the study period (1.29 ± 0.39 to 1.28 ± 0.38), resulting in a clinically significant between-group difference of 0.69 points (95% CI: 0.49-0.89, P<0.001). Lower limb muscle tone demonstrated even more pronounced between-group differences, with intervention group scores improving from 1.19 ± 0.36 to 0.53 ± 0.23, representing a 55.5% improvement in functional capacity. The between-group difference of 0.65 points (95% CI: 0.46-0.84, P<0.001) corresponded to large effect sizes for both upper (Cohen’s d = 1.76, 95% CI: 1.44-2.08) and lower (Cohen’s d = 1.94,95% CI: 1.61-2.27) extremity.

Oxygenation parameters demonstrated clinically significant between-group differences consistent with enhanced respiratory function and overall cardiopulmonary fitness. Arterial oxygen partial pressure (PaO_2_) progressively increased in the intervention group but decreased in the control group. At 8 weeks, intervention group PaO_2_ levels reached 89.3 ± 5.7 mmHg compared to 78.2 ± 6.1 mmHg in controls (between-group difference: 11.1 mmHg, 95% CI: 8.2-14.0, P<0.001), representing a clinically significant 8.5% improvement from baseline and a large effect size (Cohen’s d = 1.87,95% CI: 1.54-2.20). Arterial oxygen saturation (SaO_2_) followed similar patterns, with intervention group values of 98.0 ± 1.3% versus control group values of 94.5 ± 2.0% at 8 weeks (between-group difference: 3.5%, 95% CI: 2.4-4.6, P<0.001).

The inflammatory response, quantified using high-sensitivity C-reactive protein measurements, demonstrated notable between-group differences throughout the observation period. While both groups had comparable hs-CRP concentrations at baseline, the intervention group showed progressive reductions, reaching 4.2 ± 1.8 mg/L at 8 weeks, representing a 20.9% decrease from baseline values. Conversely, hs-CRP levels in the control group remained elevated at 5.8 ± 2.5 mg/L, resulting in a significant between-group difference of 1.6 mg/L (95% CI: 0.8-2.4, P<0.001).

Changes in body mass index provided additional data consistent with potential intervention associations in promoting healthy metabolic adaptation. The intervention group demonstrated a 6.4% increase from baseline (25.1 ± 3.1 to 26.7 ± 3.0 kg/m²), while the controls showed a modest decline (25.2 ± 2.9 to 24.4 ± 2.9 kg/m²). This 2.3 kg/m² between-group difference (95% CI: 1.2-3.4, P<0.001) may reflect improved nutritional status and enhanced lean body mass preservation in the intervention group, although the precise mechanisms cannot be determined from these data.

### Functional capacity and respiratory assessment outcomes

Functional capacity differences were evident across multiple validated assessment domains, reflecting the comprehensive benefits of the intervention. The modified Medical Research Council dyspnea scale showed progressive improvement in the intervention group, with baseline mMRC scores of 1.43 ± 0.61 decreasing to 0.61 ± 0.39 at 8 weeks, indicating a substantial reduction in perceived breathlessness during activities of daily living. Control group scores remained stable (1.49 ± 0.59 to 1.51 ± 0.56), resulting in a significant between-group difference of 0.90 points (95% CI: 0.66-1.14, P<0.001) and a large effect size (Cohen’s d = 1.82,95% CI: 1.50-2.14).

Self-efficacy for exercise, measured using the validated SEE-C scale, demonstrated marked improvement in the intervention participants, with scores increasing from 35.8 ± 4.9 to 46.0 ± 4.0, representing a 28.5% enhancement. This improvement reflects increased confidence in exercise participation and sustainable physical activity engagement, which are critical factors for long-term recovery maintenance and behavioral change sustainability. The between-group difference of 10.9 points (95% CI: 8.4-13.4, P<0.001) with a large effect size (Cohen’s d = 2.12, 95% CI: 1.77-2.47) suggested an association between intervention participation and increased confidence in exercise management.

### Categorical analysis of psychological morbidity

**Figure 5:**
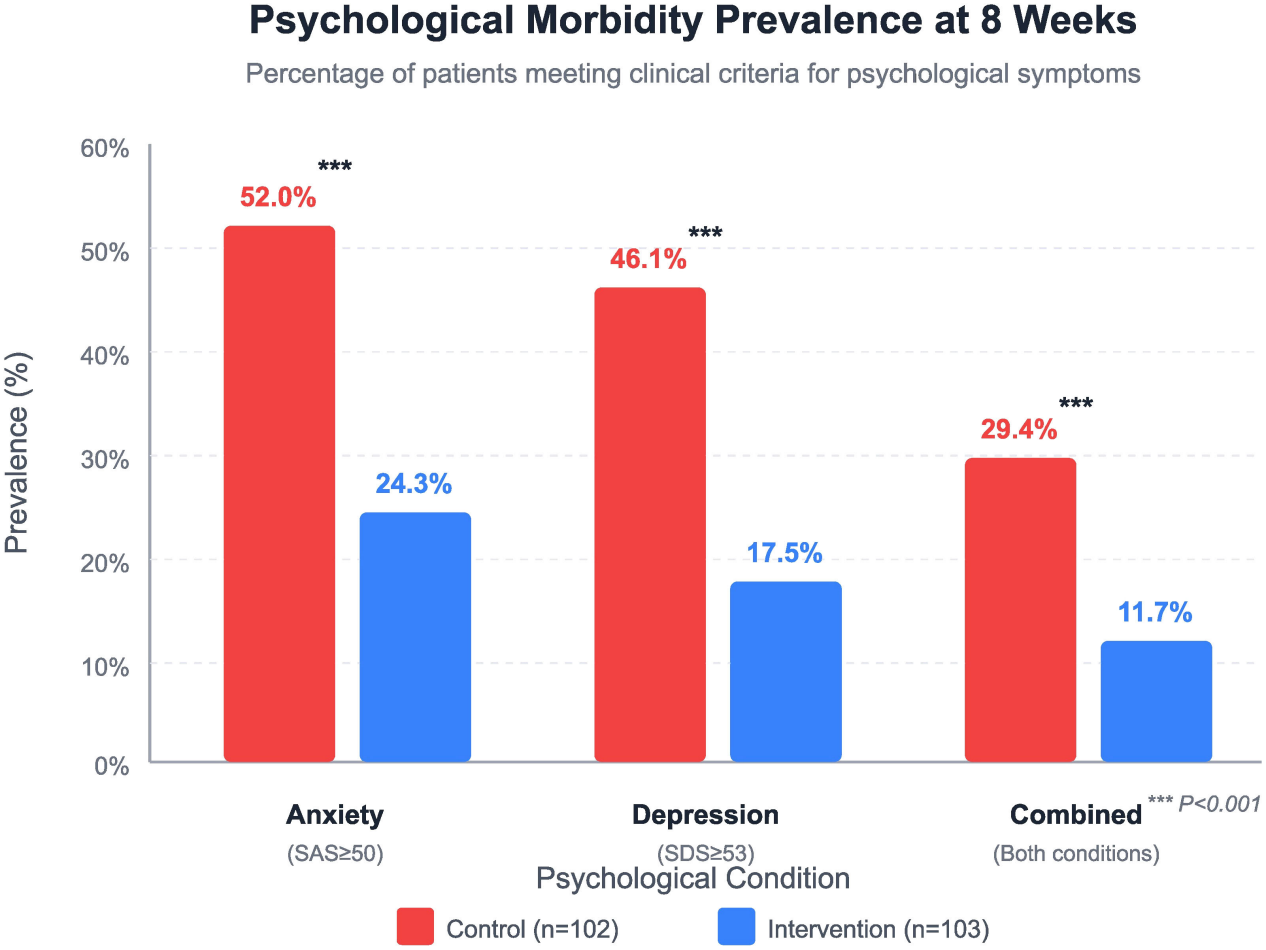
Psychological morbidity prevalence at 8 weeks post-intervention. Percentage of patients meeting the clinical criteria for anxiety (SAS≥50), depression (SDS≥53), and combined depression and anxiety at the study endpoint. Bars represent prevalence rates, with the control (red) and intervention (blue) groups shown side by side for each condition. Error bars represent the 95% confidence intervals. Statistical significance was determined using chi-square tests comparing the intervention and control groups. ***P<0.001. The intervention group demonstrated a significantly lower prevalence of psychological morbidity across all categories, with relative risk reductions of 53.3%, 62.0%, and 60.2% for anxiety, depression, and combined conditions, respectively.

Categorical analysis of the prevalence of psychological morbidity revealed substantial between-group differences in the proportion of participants who met the criteria for clinically significant psychological symptoms (**Figure 5**). At 8 weeks post-baseline, depressive symptoms (defined as SDS≥53) affected only 18 of 103 intervention participants (17.5%) compared to 47 of 102 controls (46.1%), representing a 62.0% relative risk reduction (relative risk = 0.38, 95% CI: 0.24-0.61, χ²=19.85, P<0.001, number needed to treat = 3.5) (**Table 5**).

**Table 5:** Psychological morbidity prevalence at 8 weeks post-intervention.

Condition	Intervention group n=103	Control group n=102	Relative risk (95% CI)	Relative risk reduction	χ²	P-value	NNT
Depression (SDS≥53)	18 (17.5%)	47 (46.1%)	0.38 (0.24-0.61)	62.0%	19.85	<0.001	3.5
Anxiety (SAS≥50)	25 (24.3%)	53 (52.0%)	0.47 (0.32-0.68)	53.3%	16.73	<0.001	3.6
Combined depression/anxiety	12 (11.7%)	30 (29.4%)	0.40 (0.22-0.72)	60.2%	10.48	0.001	5.7

SDS, Self-Rating Depression Scale; SAS, Self-Rating Anxiety Scale; CI, confidence interval; NNT, number needed to treat.

Similarly, anxiety symptoms (SAS≥50) were present in 25 of 103 intervention participants (24.3%) compared to 53 of 102 controls (52.0%), indicating a 53.3% relative risk reduction (relative risk = 0.47, 95% CI: 0.32-0.68, χ²=16.73, P<0.001, number needed to treat = 3.6). Combined depression and anxiety presentations occurred in 12 of 103 intervention participants (11.7%) versus 30 of 102 controls (29.4%), representing a 60.2% relative risk reduction (relative risk = 0.40, 95% CI: 0.22-0.72, χ²=10.48, P = 0.001).

### Extended follow-up and long-term outcome analysis

**Figure 6:**
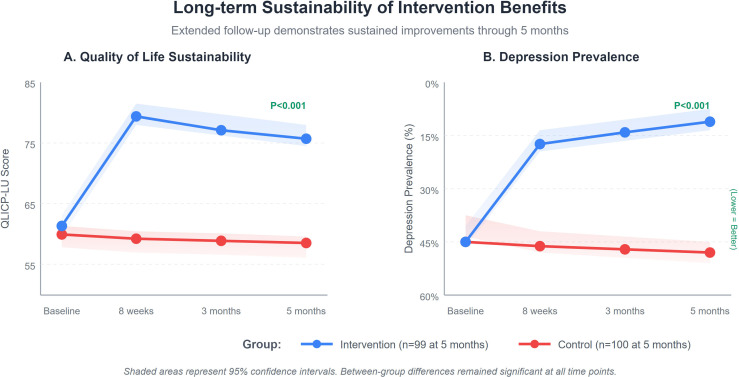
Long-term sustainability of intervention benefits. Quality of life (QLICP-LU scores) and depression prevalence trajectories from baseline to the 5-month extended follow-up period. **(A)** shows the mean QLICP-LU scores demonstrating sustained quality of life improvements in the intervention group, with a modest decline from peak values at 8 weeks but maintenance of clinically significant benefits above control levels. **(B)** shows depression prevalence (percentage meeting SDS≥53 criteria) with the Y-axis inverted so that improvement (lower prevalence) appears as an upward movement; the intervention group demonstrated a continued reduction in depression prevalence, while the control group prevalence remained elevated. Shaded areas represent 95% confidence intervals. Between-group differences remained statistically significant at all follow-up time points (P<0.001 at 5 months for both outcomes), indicating durable intervention effects that extended beyond the active treatment period. Sample sizes at 5 months: intervention, n=99; control, n=100.

Extended follow-up assessments conducted at monthly intervals for 5 months demonstrated the persistence of between-group differences across multiple outcome domains (**Figure 6**). Quality of life improvements showed sustained enhancement throughout the follow-up period, with the intervention group’s QLICP-LU scores maintaining levels significantly above the baseline and control group values. At 5 months, 97 intervention participants maintained quality of life scores of 76.2 ± 7.1 compared to 99 control group participants with scores of 59.8 ± 9.4 (between-group difference: 16.4 points, 95% CI: 12.1-20.7, P<0.001), indicating sustained clinical benefit.

Psychological symptom prevalence demonstrated persistent between-group differences throughout the extended follow-up, with intervention group participants showing continued lower rates of depression and anxiety. Longitudinal analysis revealed that depression prevalence in the intervention group decreased from 43.7% at 1 month to 11.2% at 5 months, while anxiety prevalence declined from 49.6% to 15.3% during the same period.

### Sensitivity analyses

E-value calculations indicated that to fully explain the observed association between intervention participation and QLICP-LU improvement at 8 weeks, an unmeasured confounder would need to have a risk ratio of at least 3.2, with both the intervention and the outcome, above and beyond the measured confounders (**Supplementary Table 2**). While an unmeasured confounder of this magnitude cannot be excluded, particularly given the non-randomized design, this provides context for evaluating the robustness of the findings.

Multiple imputation analyses yielded results consistent with complete-case analyses, with the imputed between-group difference in QLICP-LU of 18.8 points (95% CI: 14.3-23.3, P<0.001), supporting the consistency of findings across approaches to missing data.

## Discussion

**Figure 7:**
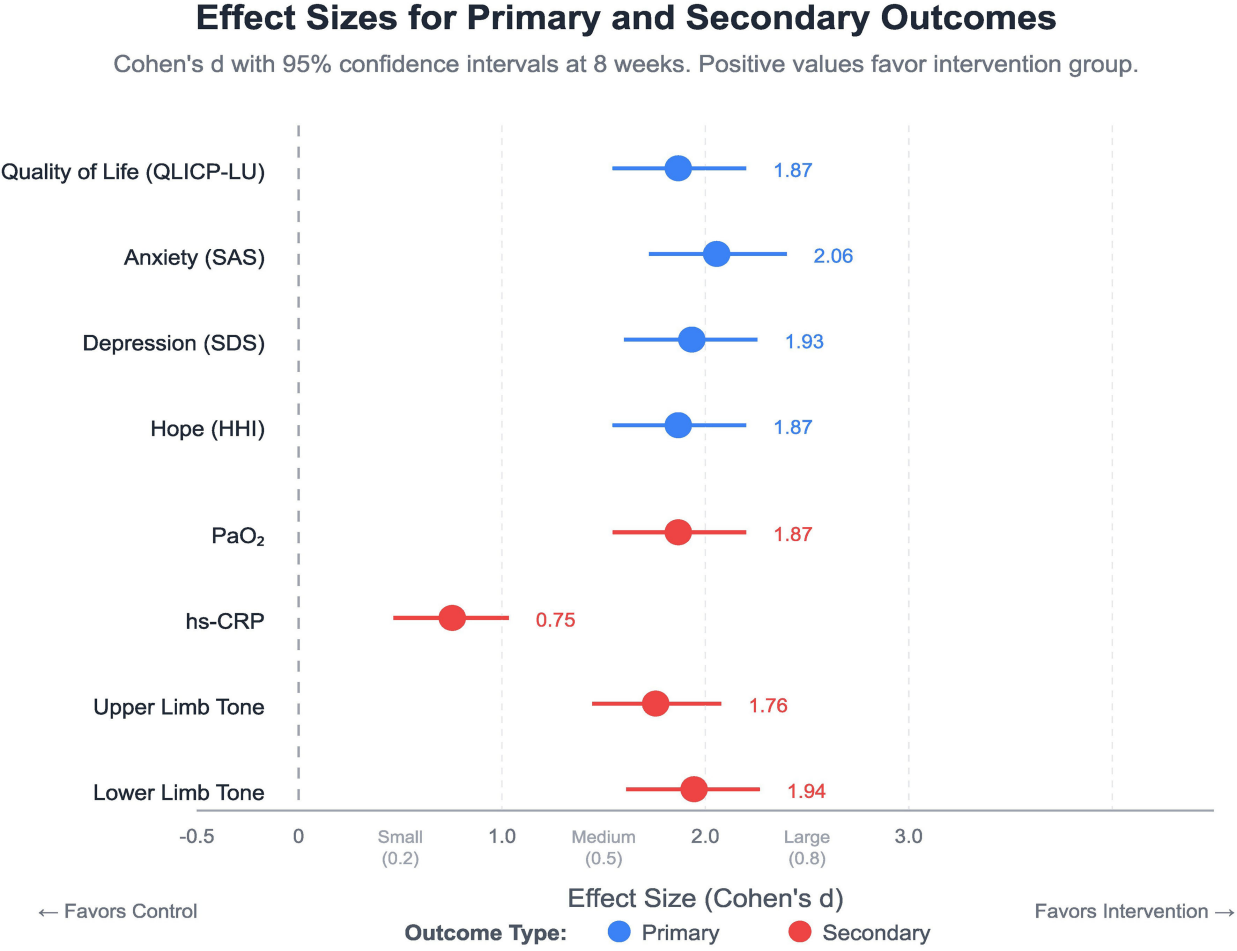
Effect sizes for primary and secondary outcomes. Forest plot displaying Cohen’s d effect sizes with 95% confidence intervals for all measured outcomes at 8 weeks post-intervention. Blue circles indicate primary outcomes (QLICP-LU, SAS, SDS, HHI); red circles indicate secondary outcomes (PaO_2_, hs-CRP, upper and lower limb muscle tone). All values were oriented such that positive values favored the intervention group. Vertical dashed reference lines indicate the conventional thresholds for small (0.2), medium (0.5), and large (0.8) effect sizes. Most outcomes demonstrated large effect sizes exceeding 0.8, with hs-CRP showing a medium-to-large effect size (d=0.75). Primary psychological outcomes (SAS and SDS) showed the largest effects. The results remained consistent in the propensity score-matched sensitivity analyses.

The present investigation provides evidence of an association between combined exercise rehabilitation and behavioral interaction nursing participation and improved multidimensional postoperative outcomes in patients with lung cancer (**Figure 7**). Our findings demonstrate that participation in the comprehensive intervention was associated with clinically meaningful differences across physiological, psychological, functional, and quality of life domains, with effect sizes consistently in the large range, and benefits sustained through five months of follow-up (1, 2). These results, which remained consistent across propensity score analyses that yielded similar findings, add to the growing body of literature supporting integrated rehabilitation approaches in cancer care.

The observed physiological differences represent clinically meaningful changes that extend beyond statistical significance to encompass functional relevance to daily living and long-term recovery trajectories. The marked between-group differences in muscle tone recovery, with 48.2% and 55.5% improvements in upper and lower extremity function, respectively, provide evidence that participation in structured, progressive exercise programs is associated with reduced deconditioning effects commonly observed after cancer surgery and extended recovery periods (8, 9). These findings align with emerging evidence from large-scale rehabilitation studies demonstrating the protective effects of early mobilization and resistance training and the maintenance of neuromuscular integrity during the oncological treatment phases (10, 11). The progressive improvement trajectory, with benefits in between-group differences becoming apparent within 2 weeks and continuing through 8 weeks, is consistent with both the rapid responsiveness of neuromuscular systems to appropriate therapeutic stimulation and the critical importance of sustained evidence-based intervention protocols for optimal recovery.

The significant differences between groups in arterial oxygenation parameters, with PaO_2_ increases of 8.5%, are consistent with the multifaceted physiological benefits of comprehensive respiratory rehabilitation in this vulnerable population (12, 13). These changes suggest improved ventilation-perfusion matching, enhanced respiratory muscle efficiency, optimized breathing patterns, and potentially increased oxygen-carrying capacity due to improved cardiovascular fitness. The magnitude of these differences substantially exceeds the typical measurement variability and approaches the clinically significant thresholds established in the pulmonary rehabilitation literature (14–16).

The 20.9% reduction in high-sensitivity C-reactive protein levels is particularly noteworthy, given the well-established relationship between chronic systemic inflammation and cancer prognosis (17, 18). Elevated CRP levels have been consistently associated with poor outcomes in lung cancer, including an increased risk of recurrence and reduced survival. The temporal pattern of CRP reduction, beginning within 2 weeks and persisting throughout the intervention period, is consistent with prior literature demonstrating exercise-associated anti-inflammatory effects (19, 20).

The psychological outcomes demonstrated the magnitude of the between-group differences achievable through integrated behavioral interventions in mental health recovery following cancer surgery, addressing a critical gap in traditional postoperative care approaches (13, 14). The 62.0% reduction in depression prevalence and 53.3% decrease in anxiety symptoms among intervention participants, while requiring cautious interpretation given the observational design, represent clinically significant improvements that exceed the efficacy of many pharmacological interventions for cancer-related psychological distress while avoiding potential side effects and drug interactions. The magnitude of these differences, combined with the corresponding increases in hope and self-efficacy measures, suggests that the behavioral interaction nursing component successfully addressed the complex psychological challenges inherent in cancer recovery.

Quality of life improvements, with QLICP-LU scores increasing by 27.3% in the intervention group, represent substantial differences in patient-reported outcomes that directly reflect the potential differences in daily functioning, symptom burden, and subjective well-being (15, 16). These improvements encompassed multiple QoL domains simultaneously, including physical functioning, emotional well-being, social relationships, and symptom management, suggesting broad-spectrum associations rather than isolated effects in specific areas of QoL. The strong correlation between hope scores and QoL measures supports theoretical models proposing hope as a fundamental mediator of adaptation and resilience in cancer populations (17, 18).

The persistence of between-group differences throughout the extended follow-up period provides important data on the durability of the observed associations. The maintenance of psychological, physiological, and QoL improvements at 5 months post-intervention among 199 of the original 205 participants may have successfully internalized behavioral changes and developed lasting coping skills (19, 20). This durability is particularly important for clinical translation because interventions with transient effects have limited practical value in chronic disease management.

The concurrent improvements observed across the physical and psychological domains are consistent with the possibility of shared underlying mechanisms, such as exercise-mediated modulation of inflammatory pathways and psychologically mediated enhancement of adaptive coping. However, we did not directly measure these pathways, and further mechanistic investigations are warranted in future studies.

Although our retrospective observational design requires appropriate caution in causal interpretation, several features of our findings merit consideration. First, the consistency of benefits across multiple independent outcome domains suggests a coherent intervention effect, rather than a spurious finding. Second, the dose-response pattern of progressive improvement over the 8-week intervention period aligned with the expected therapeutic mechanisms. Third, propensity score-matched analyses yielded results consistent with those of the unadjusted analyses, suggesting that the measured confounders did not fully account for the observed differences. Fourth, the E-value calculations indicate that unmeasured confounding of substantial magnitude would be required to explain the findings entirely. Nevertheless, the consistency of the findings across analytical approaches, while encouraging, cannot substitute for the protection against confounding that randomization provides.

Future research should explore dose-response relationships to optimize the intervention’s intensity, duration, and component combinations for different patient subgroups. Investigating moderating factors, such as tumor stage, surgical procedure type, baseline functional status, and psychosocial characteristics, could inform personalized intervention approaches and improve treatment matching. The potential of technology-enhanced delivery methods, including telehealth components, mobile health applications, and remote monitoring systems, warrants exploration to improve accessibility, reduce implementation barriers, and enhance their scalability. Prospective randomized controlled trials with rigorous methodologies are essential to establish whether the observed associations represent true causal effects and to inform clinical guidelines.

## Limitations

This study has several limitations inherent to its retrospective and observational design. The absence of randomization introduces potential selection bias, as clinicians may have preferentially recommended the rehabilitation program to patients perceived as more motivated or with a better prognosis. Although propensity score methods were employed to address the measured confounding factors, unmeasured confounders such as motivation, social support, and socioeconomic factors cannot be excluded. The very large effect sizes observed (Cohen’s d ranging from 0.75 to 2.12) are unusual for behavioral and rehabilitation interventions and may partly reflect selection bias or other unmeasured confounding factors, warranting particular caution in interpretation. Retrospective data abstraction from clinical documentation may introduce information bias despite quality assurance procedures, and the lack of blinding could contribute to performance and detection biases. The single-center design of a Chinese tertiary hospital limits the generalizability to other populations and healthcare settings, and the 5-month follow-up period precludes the assessment of longer-term outcomes, including survival and disease recurrence. Finally, the multi-component intervention prevents the determination of which specific elements are most closely associated with observed outcomes. Despite these limitations, the magnitude and consistency of the findings across multiple outcome domains, persistence of associations through extended follow-up, and robustness of results across sensitivity analyses provide a foundation for future prospective randomized controlled trials to establish causality.

## Conclusion

In conclusion, this retrospective cohort study identified an association between combined exercise rehabilitation and behavioral interaction nursing, and improved postoperative outcomes in patients with lung cancer. Participants who participated in the comprehensive rehabilitation program demonstrated improvements across physiological, psychological, functional, and quality of life domains compared with those receiving standard care; however, these findings must be interpreted with caution, given the observational design and potential for selection bias and unmeasured confounding factors. The very large observed effect sizes further underscore this need for confirmatory evidence.

Although these preliminary findings are encouraging, the non-randomized design precludes causal inference. Prospective randomized controlled trials are essential to establish whether the observed associations represent the true causal effects of the intervention. If confirmed in adequately powered RCTs, integrated rehabilitation and behavioral support programs may be a valuable addition to comprehensive oncological care for patients with lung cancer.

## Data Availability

The original contributions presented in the study are included in the article/**Supplementary Material**. Further inquiries can be directed to the corresponding author.
