# Supplementary material for: Impact of exercise rehabilitation and behavioral interaction nursing on postoperative quality of life and psychological outcomes in lung cancer patients
Source: Front Oncol. 2026 Jun 17;16:1742210. doi: 10.3389/fonc.2026.1742210 (PMC13318673; doi:10.3389/fonc.2026.1742210)
Supplement: Supplementary file 1 [file DataSheet1.docx]

**SUPPLEMENTARY MATERIALS**

**Supplementary Appendix S1: Detailed Intervention Protocols**

**S1.1 Comprehensive Exercise Prescription Details**

All exercise sessions were supervised by certified rehabilitation therapists who had completed standardized training protocols and demonstrated competency through practical examinations. Individual exercise prescriptions were developed based on baseline functional assessments, comorbidity profiles, and postoperative recovery status. The structured programme comprised three sequential components delivered according to standardized protocols, as described below.

**Respiratory Function Training**

Participants performed evidence-based diaphragmatic breathing exercises emphasizing coordinated abdominal and thoracic movement patterns to optimize tidal volume and respiratory muscle efficiency. Specific techniques included the following: (1) diaphragmatic breathing with visual biofeedback using abdominal hand placement, 10 repetitions × 3 sets; (2) pursed-lip breathing during exhalation with a 2:4-second inspiration-to-expiration ratio; and (3) incentive spirometry targeting 10 maximal inspiratory efforts per session with individualized volume goals based on predicted values. Training included pursed-lip breathing techniques designed to improve gas exchange, reduce dyspnea, and prevent atelectasis. Sessions lasted 15 minutes and were conducted twice daily under initial supervision by respiratory therapists, with progression to independent practice guided by weekly competency assessments and technique refinement.

**Upper Extremity Rehabilitation**

Progressive resistance training protocols targeted shoulder girdle musculature affected by surgical positioning and thoracic procedures. The protocol followed the American College of Sports Medicine guidelines for cancer survivors, beginning at 40–50% of estimated one-repetition maximum and progressing by 5–10% weekly as tolerated. Exercises included structured shoulder abduction, flexion, extension, and internal/external rotation movements using calibrated resistance bands and graduated weight systems (0.5–2.0 kg progression based on individual tolerance). Grip strength enhancement utilized therapeutic putty and progressive hand exercises with standardized resistance levels. Each movement was performed in supervised sets of 15 repetitions with 60-second rest periods between sets, progressing based on weekly strength assessments and symptom monitoring.

**Lower Extremity and Functional Mobility Training**

Early mobilization protocols commenced with supervised bedside standing exercises focusing on balance, coordination, and weight-bearing tolerance. Progression criteria included advancement to hallway ambulation when able to stand unsupported for 2 minutes, progression to stair climbing when able to ambulate 100 metres without rest, and outdoor walking introduced when oxygen saturation remained >92% during indoor ambulation. Progression included assisted ambulation with distance and intensity gradually increased according to individual capacity, heart rate response (target 40–60% heart rate reserve initially, progressing to 60–75%), and oxygen saturation monitoring (maintaining SpO₂ >90%). Independent indoor walking programs were implemented with target distances based on baseline functional capacity, ultimately progressing to outdoor walking when appropriate. Sessions were conducted twice daily for 15–30 minutes, with exercise intensity guided by heart rate monitoring, subjective exertion scales (modified Borg scale target 3–5 out of 10), and continuous oxygen saturation assessment.

**S1.2 Behavioural Interaction Nursing: Session-by-Session Protocols**

**Individual Psychological Counselling: Structured 8-Session CBT Protocol**

Licensed clinical psychologists with specialized oncology training provided weekly 30–45-minute individual sessions utilizing cognitive-behavioural therapy (CBT) principles specifically adapted for cancer populations. The structured 8-session protocol addressed the following topics in sequence:

Session 1 — *Psychoeducation and Treatment Rationale:* Introduction to the relationship between cancer diagnosis, surgery, and psychological distress. Normalisation of emotional responses. Collaborative goal-setting for the counselling programme. Administration of baseline psychological measures.

Session 2 — *Behavioural Activation and Activity Scheduling:* Identification of activity withdrawal patterns. Development of a graded activity schedule incorporating pleasurable and mastery activities. Monitoring of mood–activity relationships using daily diary logs.

Sessions 3–4 — *Cognitive Restructuring of Cancer-Related Fears:* Identification of automatic negative thoughts and cognitive distortions related to cancer diagnosis, recurrence fears, and catastrophic thinking. Introduction of thought records. Guided practice in generating balanced alternative appraisals. Homework assignments involving daily thought monitoring and challenging.

Session 5 — *Relaxation Training:* Instruction in progressive muscle relaxation (16 muscle group protocol adapted for postoperative limitations). Introduction to guided imagery techniques. Provision of audio recordings for independent practice. Discussion of the physiological rationale for relaxation in managing cancer-related distress.

Session 6 — *Problem-Solving Skills:* Structured problem-solving training addressing treatment-related challenges, including symptom management, communication with healthcare providers, return-to-activity planning, and social role adjustments. Use of the five-step problem-solving framework (problem identification, brainstorming, evaluation, selection, implementation and review).

Session 7 — *Communication Skills:* Strategies for effective communication with family members, healthcare providers, and social networks. Role-play exercises addressing disclosure of emotional needs, assertiveness in medical settings, and management of unsolicited advice. Discussion of caregiver burden and reciprocal support dynamics.

Session 8 — *Relapse Prevention and Maintenance Planning:* Review of skills acquired throughout the programme. Identification of early warning signs for psychological deterioration. Development of a personalised maintenance plan with specific coping strategies for anticipated future stressors. Discussion of ongoing support resources and referral pathways.

Progress was monitored using validated psychological assessment tools administered at Sessions 1, 4, and 8, with session documentation forms completed by the treating psychologist after each encounter.

**Peer Support Group Activities: Session Structure**

Structured group sessions with 6–8 participants were conducted twice weekly for 30–45 minutes each under the guidance of trained mental health professionals. The standardised group session structure was as follows:

Opening Check-In Round (5 minutes) — Each participant briefly reported on their physical and emotional status since the last meeting, with the facilitator noting themes for discussion.

Facilitated Discussion on Weekly Themes (20–25 minutes) — Discussions were organised around pre-determined weekly topics, including managing fatigue and energy conservation; communicating emotional needs with family members; navigating changes in body image and physical function; managing fear of recurrence; strategies for returning to normal activities and social roles; coping with uncertainty and maintaining hope; building and maintaining social support networks; and celebrating milestones and acknowledging progress.

Experiential Activity (10 minutes) — Activities included group relaxation exercises (guided breathing, progressive muscle relaxation), collaborative problem-solving workshops addressing common recovery challenges, therapeutic activities designed to enhance social cohesion and reduce isolation, and peer mentorship introductions pairing newly enrolled participants with programme graduates.

Closing Summary and Homework Assignment (5 minutes) — The facilitator summarised key themes, identified group strengths, and assigned a brief between-session activity (e.g., practise a relaxation technique, initiate a conversation with a family member using newly learned communication strategies).

**Comprehensive Patient and Family Education: Weekly Curriculum**

Systematic educational programmes were delivered through multimedia presentations, illustrated educational materials, and interactive learning modules. Weekly 45–60-minute sessions included both patients and family members. The 8-week curriculum was structured as follows:

Week 1 — *Understanding Your Surgery and Recovery Timeline:* Explanation of the surgical procedure performed, expected postoperative recovery trajectory, common early symptoms and their management, and an overview of the rehabilitation programme structure and expectations.

Week 2 — *Pain Management Strategies:* Multimodal pain management approaches, pharmacological and non-pharmacological options, recognising undertreated pain, communication with the medical team regarding pain concerns, and strategies for balancing pain control with functional activity.

Week 3 — *Breathing Exercises and Pulmonary Hygiene:* Rationale for respiratory rehabilitation, demonstration and supervised practice of diaphragmatic breathing and pursed-lip breathing, incentive spirometry instruction, and discussion of the importance of cough hygiene and secretion management.

Week 4 — *Nutrition for Recovery:* Nutritional requirements during postoperative recovery, protein optimisation strategies, management of treatment-related appetite changes, hydration guidance, and practical meal planning resources.

Week 5 — *Recognising Complications and When to Seek Help:* Signs and symptoms of common postoperative complications (infection, thromboembolism, respiratory deterioration), appropriate use of emergency services versus scheduled follow-up, and development of a personal emergency action plan.

Week 6 — *Emotional Adjustment and Coping Strategies:* Normalisation of psychological responses to cancer diagnosis and treatment, introduction to basic coping strategies (problem-focused and emotion-focused), family dynamics during recovery, and guidance on when to seek professional psychological support.

Week 7 — *Returning to Daily Activities and Work:* Graded return-to-activity planning, workplace accommodations and communication with employers, energy conservation techniques, and management of expectations regarding functional recovery timelines.

Week 8 — *Long-Term Survivorship and Follow-Up Care:* Ongoing surveillance schedules and their rationale, health promotion and secondary prevention strategies, long-term exercise and lifestyle recommendations, community resources and support group information, and transition planning from the rehabilitation programme to independent self-management.

Educational materials were available in multiple formats (print, digital, video) and languages as appropriate to the patient population served.

**S1.3 Therapist Training Specifications**

Prior to programme implementation, all intervention staff completed a structured training programme. Rehabilitation therapists (n=4) each possessed more than five years of oncology rehabilitation experience and completed a 20-hour supplementary training module covering the specific exercise protocols used in this programme, including safety monitoring procedures, progression criteria, and documentation requirements. Clinical psychologists (n=2) held specialised oncology training certifications and completed an 8-hour orientation to the structured CBT protocol and peer support facilitation methods used in the programme. Oncology nurses (n=6) completed a 40-hour standardised training programme encompassing the educational curriculum content, motivational interviewing techniques, fidelity monitoring procedures, and documentation standards. Competency was verified through observed practice sessions and written assessments prior to independent programme delivery.

**Supplementary Appendix S2: Extended Statistical Methods**

**S2.1 Propensity Score Model Specification**

Propensity scores representing the conditional probability of receiving the comprehensive rehabilitation intervention were estimated using a multivariable logistic regression model. The dependent variable was a binary indicator of group assignment (1 = intervention, 0 = control). The following covariates were included as independent variables based on clinical relevance and availability in the medical record: age (continuous, years), sex (binary), body mass index (continuous, kg/m²), tumour stage (binary: I–II versus III), histological type (binary: NSCLC versus SCLC), baseline QLICP-LU score (continuous), baseline SAS score (continuous), baseline SDS score (continuous), baseline PaO₂ (continuous, mmHg), and baseline mMRC dyspnoea score (continuous).

The model demonstrated adequate discrimination with a C-statistic of 0.68. The Hosmer–Lemeshow goodness-of-fit test was non-significant (P = 0.42), indicating acceptable model calibration. The distribution of propensity scores showed adequate overlap between the intervention and control groups, confirming that the positivity assumption was not substantially violated.

**S2.2 Propensity Score Matching Procedure**

One-to-one nearest-neighbour matching without replacement was performed using a caliper width of 0.2 standard deviations of the logit of the propensity score, as recommended by Austin (2011). This caliper width has been shown to eliminate approximately 98% of bias due to measured confounders under a range of scenarios. Of the 103 intervention and 102 control participants, 89 matched pairs were successfully formed. The 14 unmatched intervention participants and 13 unmatched control participants had propensity scores in regions of limited overlap. Covariate balance before and after matching was assessed using standardised mean differences; results are reported in Supplementary Table S1. After matching, all standardised mean differences were below the conventional threshold of 0.10, indicating adequate balance on all measured covariates.

**S2.3 Inverse Probability of Treatment Weighting (IPTW)**

As a complementary approach to propensity score matching, inverse probability of treatment weighting was conducted using the full cohort of 205 participants. Stabilised weights were calculated as:

For intervention participants: w = P(Treatment) / PS For control participants: w = (1 − P(Treatment)) / (1 − PS)

where PS denotes the individual propensity score and P(Treatment) denotes the marginal probability of receiving the intervention. To mitigate the influence of extreme weights, weight truncation was applied at the 1st and 99th percentiles. After truncation, the effective sample sizes were 98.2 for the intervention group and 96.7 for the control group. Covariate balance in the pseudo-population created by IPTW was verified using weighted standardised mean differences, all of which were below 0.05.

IPTW-adjusted between-group differences were estimated using weighted linear regression models with robust (sandwich) standard errors to account for the weighting. IPTW-adjusted results for primary outcomes were as follows: QLICP-LU between-group difference 18.3 points (95% CI: 13.9–22.7, P<0.001); SAS between-group difference −12.8 points (95% CI: −16.4 to −9.2, P<0.001); SDS between-group difference −11.5 points (95% CI: −15.0 to −8.0, P<0.001); HHI between-group difference 10.1 points (95% CI: 7.3–12.9, P<0.001).

**S2.4 Linear Mixed-Effects Model Specification**

Primary longitudinal analyses employed linear mixed-effects models fitted using restricted maximum likelihood estimation. The general model specification was:

Y_ij = β₀ + β₁(Group_i) + β₂(Time_j) + β₃(Group_i × Time_j) + β₄(Covariates_i) + u_i + ε_ij

where Y_ij denotes the outcome for participant i at time j, Group_i is a binary indicator of intervention assignment, Time_j is a categorical variable representing the assessment time point (baseline, 2 weeks, 4 weeks, 8 weeks), u_i is a random intercept for participant i (u_i ~ N(0, σ²_u)), and ε_ij is the residual error (ε_ij ~ N(0, σ²_ε)). Covariates included age, sex, tumour stage (I–II versus III), and the baseline value of the outcome variable.

The group-by-time interaction term (β₃) was the primary parameter of interest, representing the differential change in outcome over time between the intervention and control groups. An unstructured covariance matrix was used for the random effects. Model assumptions were evaluated through inspection of residual plots, Q–Q plots for normality of random effects, and assessment of homoscedasticity.

**S2.5 E-Value Calculations for Unmeasured Confounding**

E-values were calculated for all primary outcomes to quantify the minimum strength of association on the risk ratio scale that an unmeasured confounder would need to have with both the treatment assignment and the outcome to fully explain the observed association, conditional on the measured covariates (VanderWeele and Ding, 2017). E-values were computed using the following formula for continuous outcomes expressed as standardised mean differences:

E-value = RR + √(RR × (RR − 1))

where RR is the approximate risk ratio derived from the Cohen's d effect size using the conversion: RR ≈ exp(0.91 × d). Results are reported in Supplementary Table S2. As an interpretive guide, an E-value of 3.21 for the QLICP-LU outcome indicates that an unmeasured confounder would need to be associated with at least a 3.21-fold increase in both the probability of receiving the intervention and the probability of achieving the observed quality-of-life improvement, above and beyond the confounders already adjusted for, to reduce the observed association to the null. While unmeasured confounders of this magnitude cannot be excluded — particularly given the non-randomised design and the possibility that factors such as patient motivation, social support, and socioeconomic status may be strong predictors of both treatment selection and outcome — these E-values provide a quantitative framework for assessing sensitivity to unmeasured confounding.

**S2.6 Multiple Imputation for Missing Data**

Missing data patterns were examined and found to be less than 5% for primary outcomes at all assessment time points. Little's test for missing completely at random was non-significant (χ² = 18.4, df = 22, P = 0.68), suggesting that the missing-at-random assumption was tenable. Multiple imputation was conducted using the multivariate imputation by chained equations (MICE) procedure with m = 20 imputed datasets. The imputation model included all outcome variables at all time points, the treatment group indicator, and all covariates used in the propensity score model, in accordance with recommendations to make the imputation model at least as rich as the analytic model (White et al., 2011).

For each imputed dataset, the primary linear mixed-effects model was fitted, and parameter estimates and standard errors were combined across imputations using Rubin's rules. Imputed results were consistent with complete-case analyses: the imputed between-group difference in QLICP-LU at 8 weeks was 18.8 points (95% CI: 14.3–23.3, P<0.001), compared with 19.1 points in the complete-case analysis. Imputed results for SAS (between-group difference: −13.2, 95% CI: −16.7 to −9.7), SDS (−12.0, 95% CI: −15.6 to −8.4), and HHI (10.3, 95% CI: 7.5–13.1) were similarly concordant with complete-case findings, supporting the robustness of results to assumptions regarding missing data.

**References for Supplementary Materials**

1. Austin PC. An introduction to propensity score methods for reducing the effects of confounding in observational studies. Multivariate Behavioral Research. 2011;46(3):399-424.
2. VanderWeele TJ, Ding P. Sensitivity analysis in observational research: introducing the E-value. Annals of Internal Medicine. 2017;167(4):268-274.
3. White IR, Royston P, Wood AM. Multiple imputation using chained equations: issues and guidance for practice. Statistics in Medicine. 2011;30(4):377-399.
