# Supplementary material for: Impact of exercise rehabilitation and behavioral interaction nursing on postoperative quality of life and psychological outcomes in lung cancer patients
Source: Front Oncol. 2026 Jun 17;16:1742210. doi: 10.3389/fonc.2026.1742210 (PMC13318673; doi:10.3389/fonc.2026.1742210)
Supplement: Supplementary file 2 [file Table1.docx]

**SUPPLEMENTARY MATERIALS**

**Supplementary Table S1. Covariate Balance Before and After Propensity Score Matching**

| **Covariate** | **SMD Before Matching** | **SMD After Matching** |
| --- | --- | --- |
| Age | 0.14 | 0.04 |
| Male sex | 0.01 | 0.02 |
| BMI | 0.03 | 0.01 |
| Stage III disease | 0.01 | 0.03 |
| NSCLC histology | 0.03 | 0.02 |
| Baseline QLICP-LU | 0.14 | 0.05 |
| Baseline SAS | 0.10 | 0.03 |
| Baseline SDS | 0.09 | 0.04 |
| Baseline PaO₂ | 0.23 | 0.08 |
| Baseline mMRC | 0.10 | 0.06 |
| Baseline upper limb tone | 0.37 | 0.07 |
| Baseline lower limb tone | 0.13 | 0.05 |

*SMD: standardised mean difference. Values <0.1 indicate adequate balance. After matching, all covariates achieved adequate balance. The largest pre-matching imbalance was observed for baseline upper limb muscle tone (SMD = 0.37), which was reduced to 0.07 following matching.*

**Supplementary Table S2. Sensitivity Analyses: E-values for Primary Outcomes**

| **Outcome at 8 Weeks** | **Observed Effect Size (Cohen's d)** | **E-value for Point Estimate** | **E-value for CI Lower Bound** |
| --- | --- | --- | --- |
| QLICP-LU | 1.87 | 3.21 | 2.58 |
| SAS | 2.06 | 3.54 | 2.83 |
| SDS | 1.93 | 3.32 | 2.65 |
| HHI | 1.87 | 3.21 | 2.58 |

*E-values indicate the minimum strength of association on the risk ratio scale that an unmeasured confounder would need to have with both the intervention assignment and the outcome to fully explain the observed association, above and beyond the measured confounders included in the propensity score model. E-values for the confidence interval lower bound represent the threshold required to shift the lower bound of the 95% confidence interval to include the null. Interpretation: while confounders of this magnitude cannot be excluded in the absence of randomisation, particularly for factors such as patient motivation, social support, and socioeconomic status, these values provide a quantitative benchmark for evaluating the plausibility of unmeasured confounding as a complete explanation for the observed associations.*
